# Molecular recognition of the atypical chemokine-like peptide GPR15L by its cognate receptor GPR15

**DOI:** 10.1038/s41421-024-00698-5

**Published:** 2024-06-25

**Authors:** Zhongyuan Zhang, You Zheng, Lu Xu, Yang Yue, Kexin Xu, Fei Li, Fei Xu

**Affiliations:** 1 iHuman Institute, ShanghaiTech University, Shanghai, China; 2 School of Life Science and Technology, ShanghaiTech University, Shanghai, China; 3 Shanghai Clinical Research and Trial Center, Shanghai, China

**Keywords:** Cryoelectron microscopy, Cell signalling

Dear Editor,

The atypical chemoattractant receptor GPR15, a class-A GPCR, is significantly involved in colorectal cancer pathogenesis and the maintenance of intestinal immune homeostasis. Originally identified as a co-receptor for human immunodeficiency virus (HIV) or simian immunodeficiency virus (SIV), GPR15 regulates the targeted homing of T cells, notably FOXP3^+^ regulatory T cells (Tregs), to the lamina propria of the large intestine^1^. Moreover, GPR15 mediates Tregs homing and immunosuppression in the mouse colon^2^. Recently, a natural ligand for GPR15, C10orf99 (GPR15L), has been identified. It is a chemokine-like peptide strongly expressed in gastrointestinal tissues, confirming that the GPR15-GPR15L axis constitutes a novel signaling pathway capable of regulating intestinal homeostasis and inflammation through the migration of immune cells^3,4^.

GPR15L shares certain characteristics with the CC chemokine family. Its uniqueness lies in the C-terminal region, serving as the activation domain, distinguishing it from traditional chemokines^4^. Specifically, the 11 amino acids at the C-terminus of GPR15L (GPR15L^C11^) have been shown to exhibit potent efficacy in activating GPR15^5^.

Understanding the molecular recognition between the GPR15L–GPR15 pair and the signaling mechanism of GPR15 via its downstream Gi proteins is crucial to providing insight into the development of peptide-derived ligands and pharmaceuticals to treat intestinal disorders. To this end, we present the Cryo-EM structure of the GPR15–Gi complex bound to GPR15L^C11^ at 2.9 Å resolution (Fig. 1a, b; Supplementary Figs. S1, S2, S3, Table S1 and Materials and Methods). This structural elucidation marks a significant stride towards unraveling the intricacies of GPR15-mediated signaling and provides a solid foundation for designing and optimizing therapeutic interventions targeting this pathway.

**Fig. 1:**
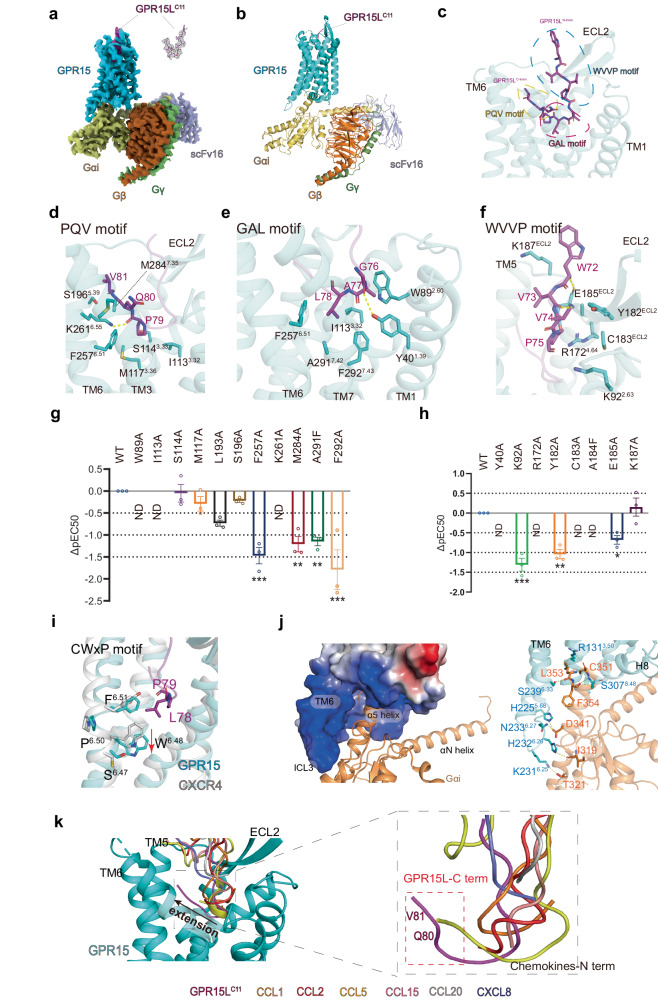
Cryo-EM structure of the GPR15L^C11^-bound GPR15–Gi complex, the mutagenesis study, and distinct binding modes of GPR15L from typical chemokines. **a**, **b** Cryo-EM map and model of the GPR15L^C11^–GPR15–Gi complex structure. **c** Three binding sites of GPR15L^C11^ in GPR15. **d**–**f** Respective interactions in the three binding sites between GPR15L^C11^ and GPR15. Yellow dashed lines denote hydrogen bonding interactions. **g**, **h** Effects of mutations in the GPR15 downstream signaling induced by GPR15L^C11^ measured by the cAMP response. Data are presented as mean ± SEM (*n* = 3 independent experiments performed in technical duplicates). All data were analyzed by two-sided, one-way ANOVA by Dunnett’s multiple test compared with the wild type (WT). *P < 0.05, **P < 0.01, and ***P < 0.001 were considered statistically significant. **i** Magnified views of the activation-related motifs within the GPR15 complex (cyan) compared to the antagonist-bound inactive CXCR4 (gray, PDB: 3OE0). Key residues are shown as sticks. Residues that are equivalent between GPR15 and CXCR4 are labeled. **j** GPR15–Gα_i_ interaction interface. GPR15 is shown in cyan and Gα_i_ in orange. **k** Structural superpositions of GPR15L^C11^-bound GPR15 with representative chemokine peptide-bound chemokine receptors; chemokines and GPR15L^C11^ are in different colors as annotated; receptors are all shown in the gray cartoon. CCR8 (PDB: 8U1U), CCR1 (PDB: 7VL9), CCR2 (PDB: 7XA3), CCR5 (PDB: 7O7F), CCR6 (PDB: 6WWZ), CXCR2 (PDB: 6LFO).

Using the Glo-Sensor assay (see Materials and Methods), we profiled the binding of GPR15L^C11^ which exhibits high-affinity recognition and activation of GPR15’s Gi pathway, albeit with ∼40 times lower potency compared to the full-length GPR15L (Supplementary Fig. S4a). The complex structure elucidates the precise positioning of GPR15L^C11^ within the receptor pocket. The peptide adopts a “V” shape and occupies a transmembrane pocket, formed by residues from transmembrane (TM) segments TM1/2, TM3/4, TM5/6/7, and the second extracellular loop (ECL2) in the extracellular side of GPR15 (Fig. 1c). We divide the binding interface into the “PQV” motif (79–81 aa), “GAL” motif (76–78 aa), and “WVVP” motif (72–75 aa), respectively, according to the natural sequence of the C-terminal 10 residues of GPR15^C11^ (Fig. 1c–f). The main chains of V81 and P79 of the “PQV” motif of GPR15L^C11^ form polar interactions with the side chains of S196^5.39^ and K261^6.55^ of GPR15, respectively (superscripts indicate B–W numbering for class-A GPCR^6^) (Fig. 1d). The main chain of G76 of the “GAL” motif forms a hydrogen bond with Y40^1.39^ in the receptor (Fig. 1e). The functional assay shows that K261^6.55^A and Y40^1.39^A mutations completely abolished ligand-induced receptor activation (Fig. 1g, h; Supplementary Fig. S4b), suggesting that this hydrogen bonding interaction plays a key role in receptor recognition (Fig. 1d, e; Supplementary Fig. S4b). In the “WVVP” motif, interactions with the receptor include the main chain of V73 and W82 forming hydrogen bonds with E185^ECL2^, stabilizing the conformation of the ligand and the extracellular portion of the receptor (Fig. 1f). Additionally, a panel of single mutations including W89^2.60^A, K92^2.63^A, I113^3.32^A, R172^4.64^A, Y182^ECL2^A, C183 ^ECL2^A, E185 ^ECL2^A, K187 ^ECL2^A, F257^6.51^A, A291^7.42^F and F292^7.43^A all markedly reduce the activation of GPR15 (Fig. 1g, h; Supplementary Fig. S4 and Table S2). Specifically, K92 of GPR15 makes polar interaction with P75 of GPR15L^C11^, while M284 and F292 engage in hydrophobic interactions with the ligand. Mutations at these positions disrupt critical interactions with the ligand, resulting in reduced activation of the receptor. Additionally, the mutation of A291F introduces a clash with L78 on the ligand, further impairing receptor activation. These results confirm the important roles of the hydrogen bonding and hydrophobic interactions in this region.

To elucidate the conformational changes following the activation of GPR15 by GPR15L^C11^, we aligned the structure of GPR15–Gi with inactive CXCR4 (PDB: 3OE0). The binding of GPR15L^C11^ engages in a hydrophobic interaction with F257^6.51^ of GPR15, inducing its downward shift (Fig. 1i). Accordingly, F257^6.51^A mutation profoundly reduces the activation of GPR15 (Fig. 1g). This conformational change is accompanied by a downward rotational movement of toggle switch residue W254^6.48^ (Fig. 1i). The interaction of GPR15L^C11^ with GPR15 also leads to a twisting movement of TM7 towards TM6. This is primarily driven by the hydrophobic side chains of Q80 and L78 from GPR15L^C11^ interacting with M284^7.35^, A291^7.42^, and F292^7.43^ of TM7, influencing the movement of TM7 along with the displacement of TM5 and the outward movement of the extracellular part of TM6 (Fig. 1d, e; Supplementary Fig. S5a**)**. Subsequent conformational alterations include rearrangement of the P^5.50^V^3.40^F^6.44^ motif, an outward kink in the TM6 intracellular domain, collapse of the Na^+^ pocket, rearrangements in the DR^3.50^Y motif and the conserved NPxxY motif (Supplementary Fig. S6)^6^. These conformational changes ultimately result in the opening of the intracellular cavity to accommodate the G protein, consistent with the canonical activation mechanism observed in class-A GPCRs^6^. The interaction between Gi proteins and GPR15 is similar to that of other GPCR–Gi complexes. The C-terminal residues of Gαi protein, including I319, T321, D341, C351, L353, and F354, engage in polar interactions with residues on TM3, TM5, TM6, and H8 of GPR15 (Fig. 1j).

GPR15L shares functional similarities with chemokines, notably in mediating the migration of immune cells^7^. The molecular recognition of chemokines for their cognate chemokine receptors was previously investigated in detail^8–14^. In our GPR15L–GPR15–Gi structure, the ligand portion within the pocket consists of 10 residues, whereas pockets occupied by other chemokines typically span only 5–8 residues, supporting a highly specific receptor recognition for GPR15L. Compared with other chemokines, GPR15L extends further within the receptor pocket and is positioned closer to TM5 and TM6 (Fig. 1k). A notable difference in receptor recognition between GPR15L and other chemokines lies in the fact that while GPR15L employs its C-terminal 10 amino acids to recognize GPR15, all other chemokine–receptor interactions are primarily mediated by the chemokine’s N-terminus (Fig. 1k). Due to the lack of conservation in the N-terminus among chemokines, various chemokines can activate different chemokine receptors. However, GPR15L exclusively activates GPR15, differentiating it from typical chemokines^3^. In our structure, we noted a unique positioning of GPR15L that induces movement in TM5, TM6, and TM7 of GPR15 relative to other chemokine receptors (Supplementary Fig. S5a). This movement arises from the unique interaction pattern by two residues, Q80 and V81, in GPR15L, potentially resulting in clashes with corresponding positions in other chemokine receptors (Fig. 1k). This observation suggests that GPR15L may exclusively activate GPR15 through a unique recognition mechanism, emphasizing the specificity of their interaction. Moreover, the side chain of L78 of GPR15L extends into the pocket, making a distance of approximately 5.5 Å with the W^6.48^ position. This distance is notably shorter than in other chemokines with known structures (Supplementary Fig. S5b). In conclusion, GPR15L employs a distinctive recognition mode through a “V-shaped” peptide at its C-terminus to occupy a deep-binding pocket within GPR15, distinguishing it from other chemokines (Supplementary Fig. S5b).

Given the pivotal role of GPR15, particularly as a co-receptor for HIV/SIV and its significant involvement in colorectal cancer as well as in regulating colonic inflammation and immune homeostasis, the imperative for drug design targeting GPR15 is undeniable. In this study, we analyzed the structure of the complexes formed between GPR15 and the endogenous ligand GPR15L^C11^ coupled to the downstream Gi proteins, thereby gaining a deep understanding of the ligand recognition and activation mechanisms of GPR15. This holds significant implications for developing peptide and small molecule drugs targeting GPR15. Furthermore, the 10-aa V-shaped conformation of GPR15L within the pocket suggested that this peptide sequence could be potentially grafted to the variable regions of an antibody fragment, which may represent a favorable direction in drug design.

The structural analysis of this study does not encompass the N-terminal region of GPR15L. While the full-length ligand demonstrates superior activation potency compared to GPR15L^C11^ (Supplementary Fig. S4a), prior research has highlighted the pivotal role of the 11 amino acids located in the C-terminus for receptor recognition and activation^3,5,15^. We hypothesized that the N-terminal portion might participate in interactions with regions outside the orthosteric pocket of the receptor (pertaining to the interaction between chemokine C-termini and their receptors), potentially influencing ligand activation potency to some extent. However, truncating the C-terminal 11 amino acids from full-length GPR15L renders the peptide incapable of activating the receptor^3,15^, thereby affirming the essential role of interactions between GPR15L^C11^ and the orthosteric pocket of GPR15. Nonetheless, further investigations into the full-length structure of GPR15L are warranted to comprehensively elucidate its functional significance.

### Supplementary information

Supplementary Information

## Data Availability

The atomic coordinates and the electron microscopy maps of the GPR15L^C11^–GPR15–Gi have been deposited in the Protein Data Bank (PDB) under accession code 8ZQE, and Electron Microscopy Data Bank (EMDB) under accession code EMD-60384.
