# Supplementary material for: Molecular recognition of the atypical chemokine-like peptide GPR15L by its cognate receptor GPR15
Source: Cell Discov. 2024 Jun 25;10:69. doi: 10.1038/s41421-024-00698-5 (PMC11199581; doi:10.1038/s41421-024-00698-5)
Supplement: Supplementary file 1 — Supplementary Information [file 41421_2024_698_MOESM1_ESM.pdf]

Molecular recognition of the atypical chemokine-like peptide GPR15L by its cognate receptor GPR15

Zhongyuan Zhang[1,2,3], You Zheng[1,3], Lu Xu[1], Yang Yue[1], Kexin Xu[1,2], Fei Li[1], Fei Xu[1,2,4]

[1]iHuman Institute, ShanghaiTech University, Shanghai, China.

[2]School of Life Science and Technology, ShanghaiTech University, Shanghai, China.

[3]These authors contributed equally to this work.

[4]Correspondence: xufei@shanghaitech.edu.cn (Fei Xu)

Materials and Methods

Molecular cloning of GPR15 construct

The human wild type GPR15 gene (1-360) was subcloned into the expression vector pFastBac1 vector with an N-terminal HA-Flag tag, followed by a 10x His tag and three human rhinovirus 3C (HRV3C) cleavage site. Thermostabilized apocytochrome b_{562}RIL (BRIL)[1] was inserted into the N-terminal of KOR, a MBP was inserted between human rhinovirus 3C (HRV3C) protease site and 10x His tag. A dominant-negative bovine DNGαi1 (Gαi1_3M) with three mutations (S47N, G203A and A326S)[2] was generated by site-directed mutagenesis to decrease the affinity of nucleotide binding and limit G protein dissociation for stable receptor-Gi complex. Gβ1 and Gγ2 are cloned into a pFastBac Dual vector.

Expression, complex formation and purification

GPR15, DNGαi and Gβ1γ2 were co-expressed in Hi5 insect cells (Invitrogen) using Bac-to-Bac baculovirus expression system (Thermo Fisher). Cell cultures were grown in ESF 921 medium (Expression system) to a density of 2×10^6 per mL with two separate virus preparations for GPR15, DNGαi and Gβ1γ2 at a ratio of 1:1.3:1.5. The infected cells were cultured at 27 °C for 48 h before collection by centrifugation and the cell pellets were stored at -80 °C for future use.

For the purification of GPR15L^{C11}-GPR15-Gi-scFv16 complex, GPR15L^{C11} (WVVPGALPQV) was synthesized by GenScript using solid-phase peptide synthesis, the purity of the synthesized peptide was determined to be 95%, cell pellets from 1L culture were thawed at room temperature and resuspended in low salt buffer containing 20 mM HEPES pH 7.4, 100 mM NaCl, 5 mM $CaCl_2$, 5 mM $MgCl_2$, 10% glycerol, protease inhibitor cocktail (Thermo Fisher). The GPR15-Gi complex were formed on membrane in presence of 10 μM GPR15L^{C11} and treated with apyrase (20 mU mL^{-1}, NEB), followed by incubation overnight at 4 °C. Cell membranes were collected by ultra-centrifugation at 100,000 x g for 40 min. The membranes were resuspended and then incubated with 5 μM GPR15L^{C11}, 2 mg ml^{-1} iodoacetamide (Sigma), 20 mU mL^{-1} apyrase at 4 °C for 0.5 h. The protein was extracted from the membrane by 20 mM HEPES, pH 7.4, 100 mM NaCl, 1%(w/v) lauryl maltose neopentyl glycol (LMNG, Anatrace), 0.2% (w/v) cholesteryl hemisuccinate TRIS salt (CHS, Anatrace) and stirred for 2 h at 4°C. The supernatant was isolated by centrifugation at 30000 rpm for 30 min and then incubated overnight at 4 °C with pre-equilibrated TALON IMAC resin (Clontech). After batch binding, the TALON IMAC resin with immobilized protein complex was manually loaded onto a gravity flow column. The TALON IMAC resin was washed with 10 column volumes of 20 mM HEPES, pH 7.4, 100 mM NaCl, 5 mM $CaCl_2$, 5 mM $MgCl_2$, 10% glycerol, 30 mM imidazole, 0.1% LMNG (w/v), 0.02% CHS (w/v) , 20 μM GPR15L^{C11}, and wash again with the buffer containing 0.05% LMNG (w/v), 0.01% CHS (w/v), 50 mM imidazole, 10 μM GPR15L^{C11} and elute with the buffer containing 0.01% LMNG (w/v), 0.005% CHS (w/v), 300 mM imidazole, 5 μM GPR15L^{C11}. The eluted protein was incubated with 200μg of scFv16 at 4 °C for another 0.5 h. The mixture was then purified by SEC using a Superdex 200 10/300 GL column (GE healthcare) in 20 mM HEPES, pH 7.4, 100 mM NaCl, 0.00075% (w/v) LMNG, 0.00025% (w/v) GDN, 0.00015% (w/v) CHS and 2 μM GPR15L^{C11}. The fractions of monomeric complex were

collected and concentrated to 2.5-3.5 mg mL^{-1} for electron microscopy experiments.

Cryo-EM grid preparation and data collection

For cryo-EM grid preparation, 3.5 μL of $GPR15L^{C11}$-GPR15-Gi-scFv16 complex at the concentration of 3.0 mg mL^{-1} was applied individually to EM grids (Quantifoil, 300 mesh Au R1.2/1.3) that were glow-discharged for 45s, which were blotted for 4s under 100% humidity at 4 °C before being plunged into liquid ethane cooled by liquid nitrogen using a Mark IV Vitrobot (FEI). Protein concentration was determined by absorbance at 280 nm using a Nanodrop 2000 Spectrophotometer (Thermo Fisher Scientific). The grids were loaded onto a Titan Krios electron microscope operated at 300 kV accelerating voltage equipped with Gatan K3 summit direct electron camera (Gatan) with a Gatan Qutantum energy filter. Images of all datasets were recorded with SerialEM software in EFTEM nanoprobe mode a 70 mm C2 aperture, at a calibrated magnification of 105,000 corresponding to a magnified pixel size of 0.832 Å and a defocus range of -1.1 to -2.2 μm. The slit width for zero loss peak was 20 eV. Each movie was comprised of 40 frames with a total dose of 60 electrons per Å2, exposure time was 2.0 s with the dose rate of 20 e^-/pixel/s.

Image processing

All dose-fractioned images were motion-corrected and dose-weighted by MotionCorr2 software[3] and their contrast transfer functions were estimated by patch CTF estimation in cryoSPARC[4]. For $GPR15L^{C11}$-GPR15-Gi dataset, 300 images were selected automatically to do blob picking and 132,215 particles were picked and extracted, then 2D classification with 200 classes to sort the dataset was performed in cryoSPARC v.4.5.1 and 26 classes with 23,237 particles were selected as training data to generate a deep learning model using topaz. The deep picker was performed to all 4,198 images using topaz with the pretrained model, and a total of 2,097,368 particles were autopicked and used in 2D classification. 616,647 particle projections were then selected for the subsequent 3D heterogeneous refinement in cryoSPARC. After the final round of 3D heterogeneous refinement from 547,851 particle projections, a selected subset containing 198,351 projections that displayed fine structural details were processed for high-resolution refinement, including non-uniform refinement and local refinement, resulting in the final map with an overall resolution of 2.89 Å. The overall resolution was estimated on the basis of gold-standard Fourier shell correlation of independently refined half maps and the 0.143 cutoff criterion. Local resolution was estimated in cryoSPARC using default parameters. The density map underwent automatic masking and local sharpening in DeepEMhancer to optimize local density.

Model building, refinement, and validation

For the $GPR15L^{C11}$-GPR15-Gi complex, the Gi protein from the C3aR1-Gi complex structure[5] (8HK3) was used as starting models for model building and refinement against the electron density map of Gi and scFv16. The cryo-EM model was docked into the electron microscopy density map using Chimera and further manually built in Coot-0.9.6[6] with the guidance of the cryo-EM map. Structure refinement was further performed using phenix.real_space_refine application in real space with secondary structure and geometry restraints, in combination with

manually building. UCSF Chimera[7], Chimera X[8], and PyMOL (Schrödinger) were used to prepare the structural figures in the paper.

cAMP functional assay

To determine GPR15 mediated change of cAMP production, we use split luciferase based Glosensor cAMP[9] biosensor technology. HEK293T cells were cultured in 6-well plate at a density of 4×105 cells/well for 24h, and then the cells were transiently transfected with 1μg of plasmids, with 500ng pGloSensor™-22Fa and 500ng GPR15 (or GPR15 mutation) with Lipo2000 transfection reagent. After transfection for 24h, cells were seeded into poly-D-lysine-coated 384-well culture plates in DMEM with 1% dFBS. Cells were grown overnight before incubation in 20mg/ml luciferin which diluted in HBSS for 1h at 37°C (no CO_2). To measure the 11-GPR15L activity, 10μL 4x solution was added with serial concentration and reacted for 15 min. The plates for the Gi assay were diluted by adding 10 μL isoproterenol (Sigma) at a final concentration of 200 nM, paused for 15 min. Luminescence signals were measured using an EnVision plate reader.

References

1. Chun, E., et al., *Fusion Partner Toolchest for the Stabilization and Crystallization of G Protein-Coupled Receptors.* Structure, 2012. **20**(6): p. 967-976.
2. Liang, Y.L., et al., *Dominant Negative G Proteins Enhance Formation and Purification of Agonist-GPCR-G Protein Complexes for Structure Determination.* ACS Pharmacol Transl Sci, 2018. **1**(1): p. 12-20.
3. Zheng, S.Q., et al., *MotionCor2: anisotropic correction of beam-induced motion for improved cryo-electron microscopy.* Nature Methods, 2017. **14**(4): p. 331-332.
4. Punjani, A., et al., *cryoSPARC: algorithms for rapid unsupervised cryo-EM structure determination.* Nature Methods, 2017. **14**(3): p. 290-+.
5. Wang, Y., et al., *Revealing the signaling of complement receptors C3aR and C5aR1 by anaphylatoxins.* Nature Chemical Biology, 2023. **19**(11): p. 1351-+.
6. Emsley, P. and K. Cowtan, *:: model-building tools for molecular graphics.* Acta Crystallographica Section D-Structural Biology, 2004. **60**: p. 2126-2132.
7. Pettersen, E.F., et al., *UCSF chimera - A visualization system for exploratory research and analysis.* Journal of Computational Chemistry, 2004. **25**(13): p. 1605-1612.
8. Pettersen, E.F., et al., *UCSF ChimeraX: Structure visualization for researchers, educators, and developers.* Protein Science, 2021. **30**(1): p. 70-82.
9. Wang, F.I., et al., *Luciferase-based GloSensor™ cAMP assay: Temperature optimization and application to cell-based kinetic studies.* Methods, 2022. **203**: p. 249-258.

a b

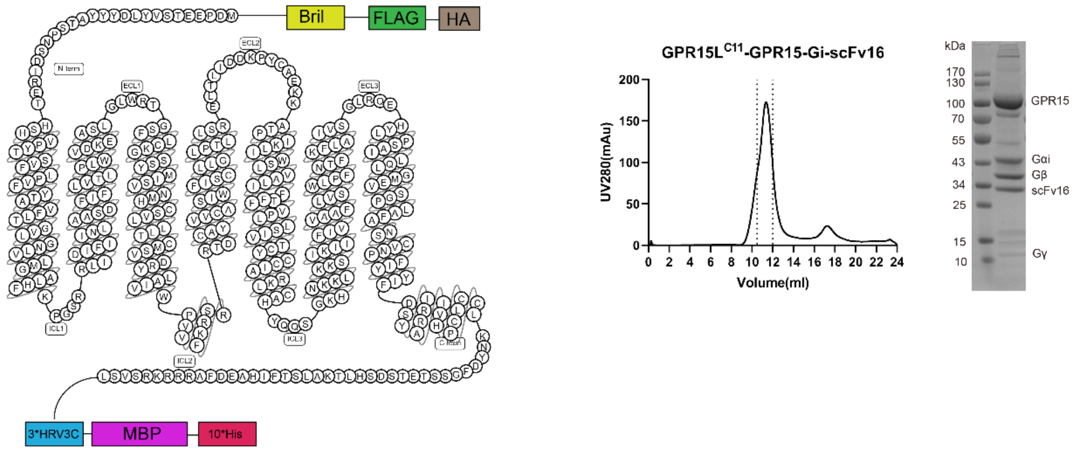

Supplementary Fig. S1. Biochemical characterization of GPR15 and the protein complex. **a** The snakeplot of full-length GPR15 construct. The N-terminus of GPR15 was fused with a HA tag, FLAG tag and Bril, the C-terminus of GPR15 was fused with MBP to enhance expression. **b** Size exclusion chromatography profile (left) and SDS-PAGE analysis (right) of the GPR15L^{C11}-GPR15-DNGi-scFv16 complex.

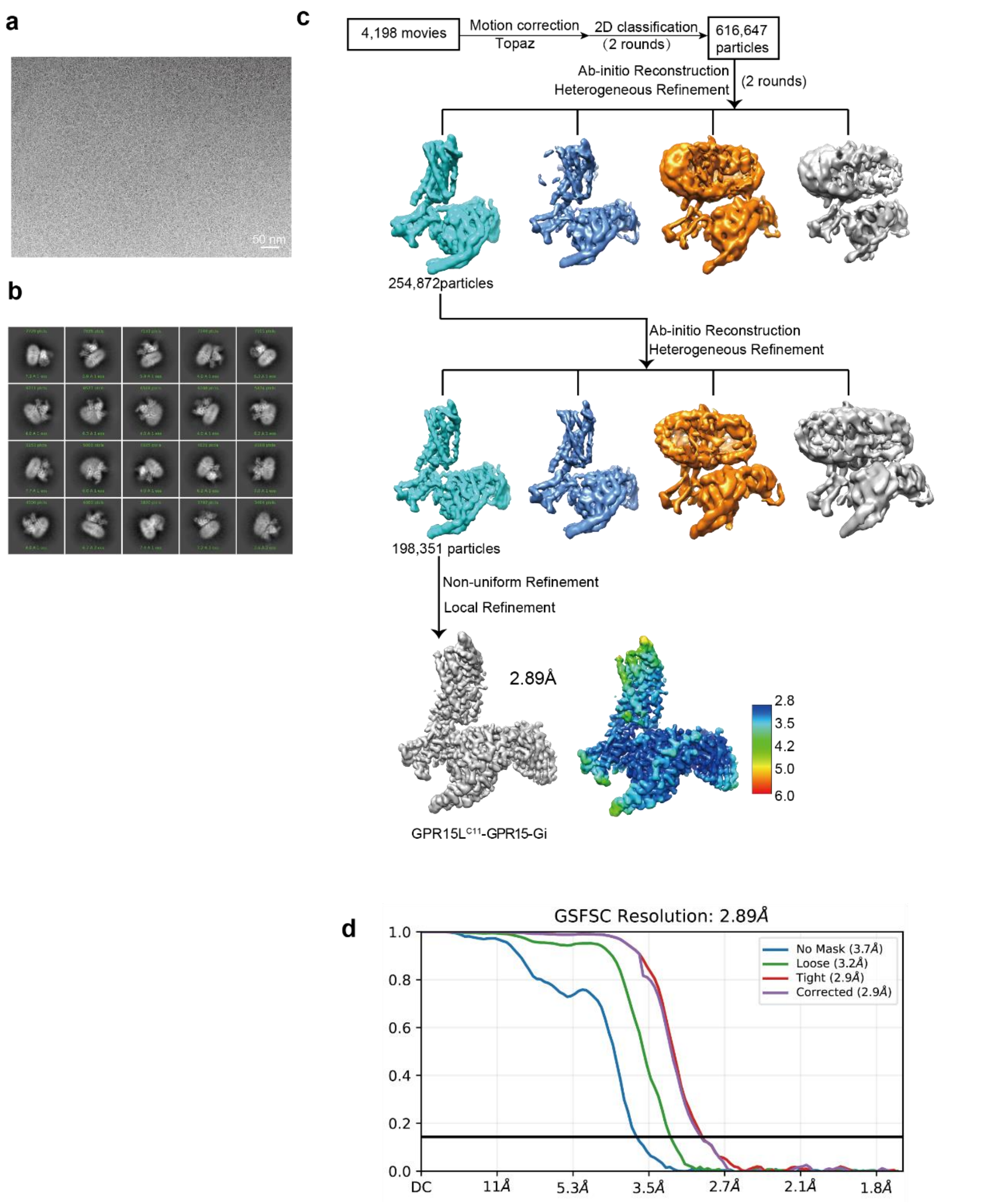

Supplementary Fig. S2. Structure determination of the GPR15L^{C11}-GPR15-Gi-scFv16 complex. a Cyro-EM raw image of GPR15L^{C11}-GPR15-Gi-scFv16 complex. **b** Cyro-EM 2D classification averages of GPR15L^{C11}-GPR15-Gi-scFv16 complex. **c** Cryo-EM data processing flowchart of the GPR15L^{C11}-GPR15-Gi complex. **d** The global resolution of the final processed density map estimated at the FSC = 0.143 is 2.9 Å.

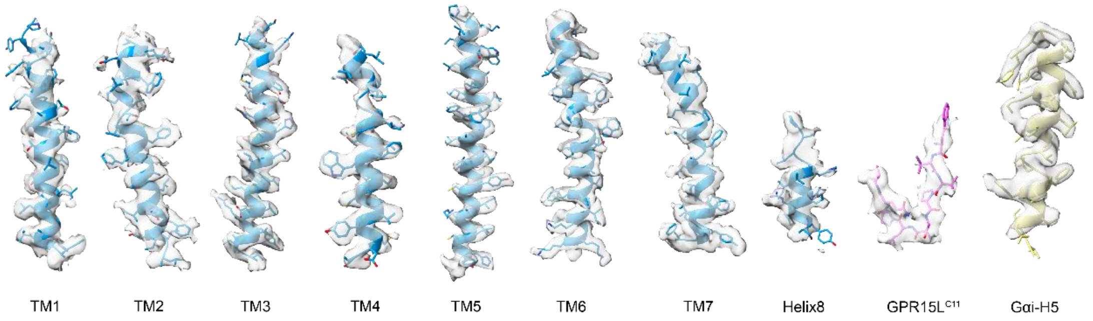

Supplementary Fig. S3. Cryo-EM map quality assessment. EM density maps of transmembrane helices TM1-TM7 and helix 8 of GPR15, GPR15L^{C11} and α5 helix of Gαi.

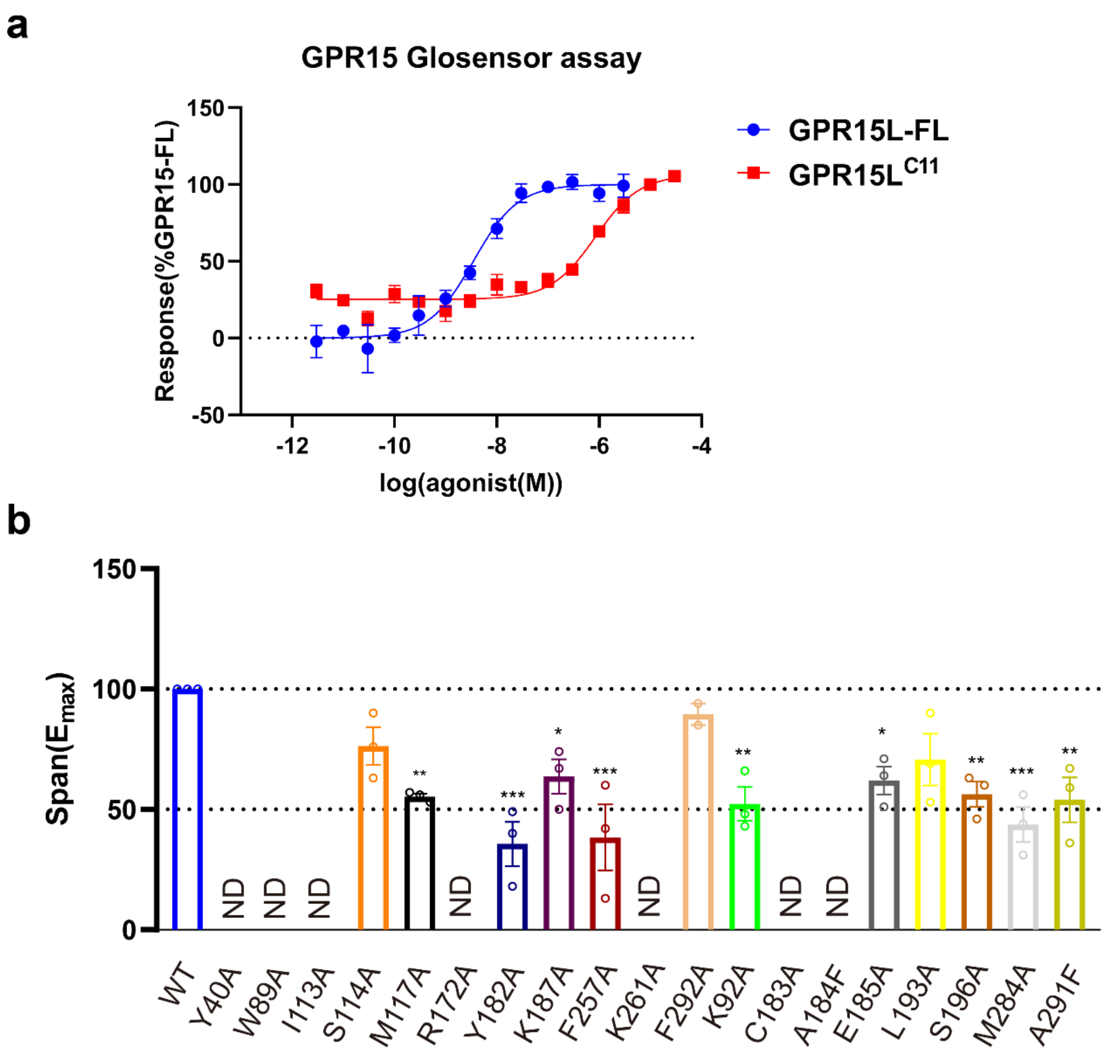

Supplementary Fig. S4. Functional characterization of GPR15 mutations. a The dose-dependent response curves of GPR15 activated by GPR15L-FL or GPR15L^{C11} using GloSensor cAMP assay. **b** Effects of mutations in the GPR15 signaling efficacy (E_{max}) induced by GPR15L^{C11}. Data are presented as means ± s.e.m. of N = 3 independent experiments performed in technical duplicates. All data were analyzed by two-sided, one-way ANOVA by Dunnett's multiple test compared with WT. *$P < 0.05$, **$P < 0.01$ and ***$P < 0.001$ were considered statistically significant.

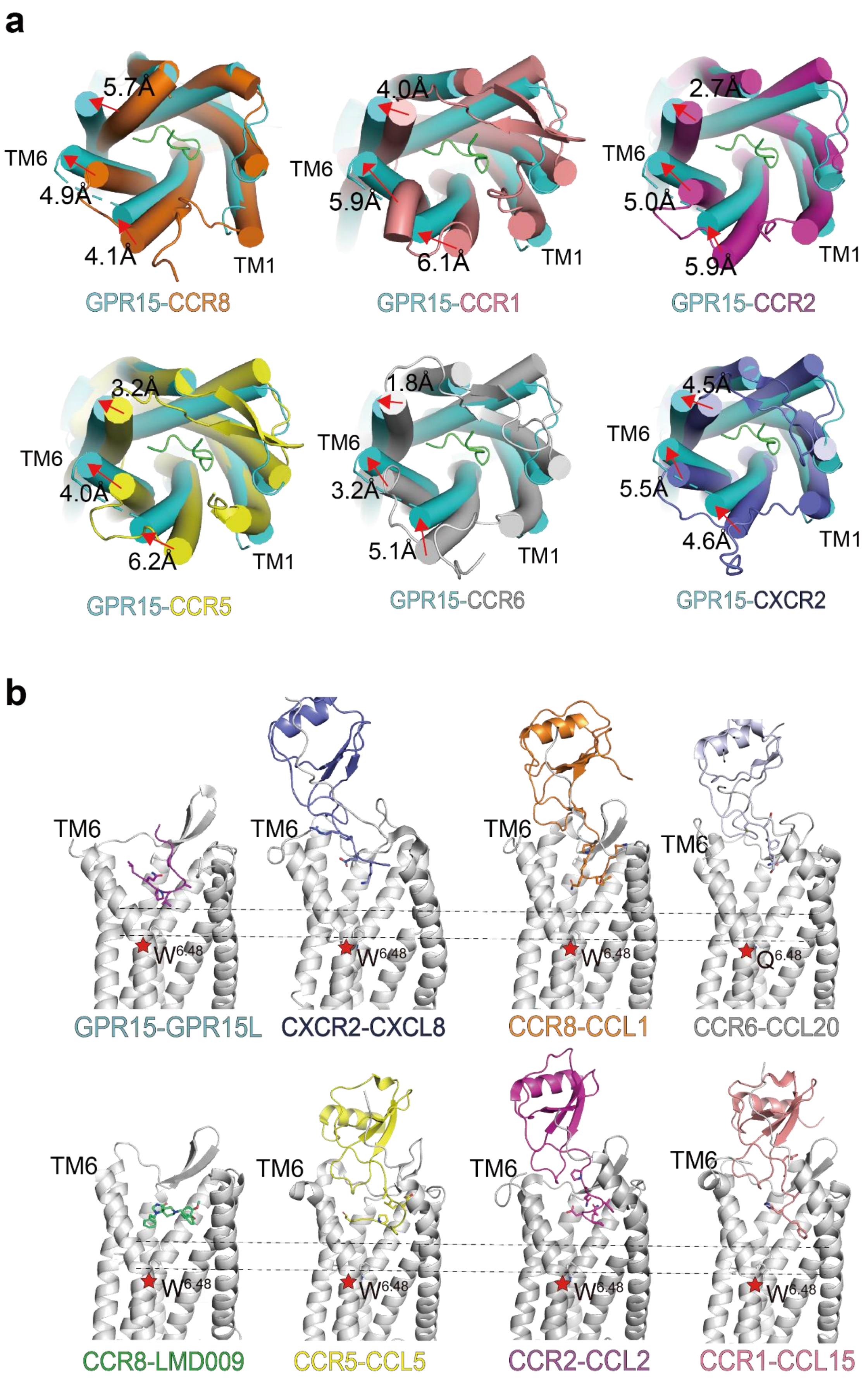
a
5.7Å
TM6
4.9Å
4.1Å
TM1
GPR15-CCR8
4.0Å
TM6
5.9Å
6.1Å
TM1
GPR15-CCR1
2.7Å
TM6
5.0Å
5.9Å
TM1
GPR15-CCR2
3.2Å
TM6
4.0Å
6.2Å
TM1
GPR15-CCR5
1.8Å
TM6
3.2Å
5.1Å
TM1
GPR15-CCR6
4.5Å
TM6
5.5Å
4.6Å
TM1
GPR15-CXCR2
b
TM6
W6.48
GPR15-GPR15L
TM6
W6.48
CXCR2-CXCL8
TM6
W6.48
CCR8-CCL1
TM6
Q6.48
CCR6-CCL20
TM6
W6.48
CCR8-LMD009
TM6
W6.48
CCR5-CCL5
TM6
W6.48
CCR2-CCL2
TM6
W6.48
CCR1-CCL15

Supplementary Fig. S5. Structural comparison of GPR15L^{C11}-bound GPR15 with chemokine-bound chemokine receptors. a Top view of GPR15 helical arrangement in comparison to each CCR-family receptor; The movements of TM5, TM6, TM7 are indicated. CCR8 (PDB: 8U1U), CCR1 (PDB: 7VL9), CCR2 (PDB: 7XA3), CCR5 (PDB: 7O7F), CCR6 (PDB: 6WWZ), CXCR2 (PDB: 6LFO). **b** Comparison of the insertion depth of the GPR15L into the GPR15 pocket with other chemokine peptides (or small molecule LMD009) in their respective chemokine receptor pockets. Stars denote the conserved tryptophan residue at position 6.48.

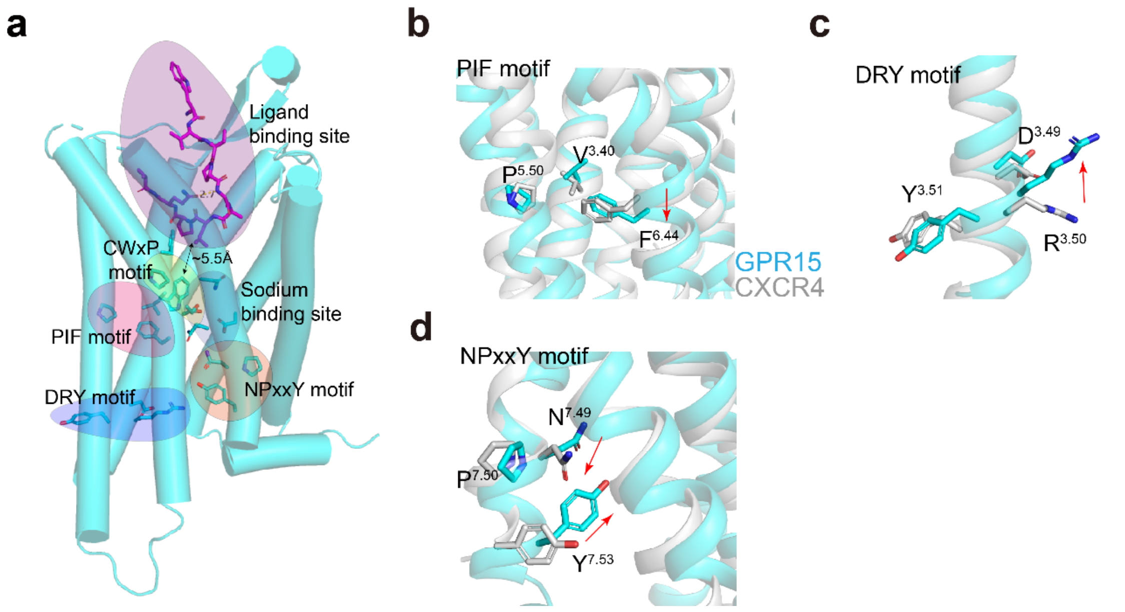

Supplementary Fig. S6. Activation mechanism of GPR15 by GPR15L^{C11}. a Ligand-induced global changes in receptor motif conformation. Motifs within GPR15, shown as coloured patches (magenta, ligand binding site; yellow, CWxP motif; red, PIF motif; purple, sodium site; orange, NPxxY motif; blue, DRY motif). **b-d** Magnified views of these motifs within the GPR15 complex (cyan) and the antagonist-bound inactive CXCR4 (grey). Each residue is shown as a stick model. Key residues that are equivalent between GPR15 and CXCR4 are labeled. The conformational changes are shown around (**b)** the PIF motif, (**c)** the DRY motif and (**d)** the NPxxY motif.

Supplementary Table S1. Cryo-EM data collection, refinement, and validation statistics

Table S1. Cryo-EM data collection, refinement, and validation statistics

	$GPR15L^{C11}$-GPR15-Gi-scFv16
Data collection and processing	
Magnification	105,000
Voltage (kV)	300
Pixel size (Å)	0.832
Electron exposure (e–/$Å^2$)	60
Defocus range (μm)	0.7-2.0
Symmetry imposed	C1
Initial particle images (no.)	752,735
Final particle images (no.)	198,351
Map resolution (Å)<br>FSC threshold	2.89<br>0.143
Refinement	
Initial model used<br>(PDB code)	8HK2<br>AlphaFlodPDB: P49685
Map resolution (Å)<br>FSC threshold	2.89<br>0.143
Map sharpening B factor ($Å^2$)	-129.6
Model composition<br>Non-hydrogen atoms<br>Protein residues<br>Ligands	 8,767<br>1,131<br>$GPR15L^{C11}$
Average B factors ($Å^2$)<br>Protein<br>Ligand	 76.7<br>---
R.m.s. deviations<br>Bond lengths (Å)<br>Bond angles (°)	 0.004<br>0.715
Validation<br>MolProbity score<br>Clash score<br>Poor rotamers (%)	 1.91<br>12.16<br>0.21
Ramachandran plot<br>Favored (%)<br>Allowed (%)<br>Disallowed (%)	 95.50<br>4.50<br>0.0

Supplementary Table S2. Summary of cAMP functional assay for the tested GPR15 mutants

	pEC_{50}	E_{max}(%)
WT	6.23 ±0.08	100 ±0.00
S114A	6.20 ±0.12	76.33 ±7.80
M117A	5.94 ±0.09	55.33 ±1.20 **
Y182A	5.188 ±0.04	35.67 ±9.21 ***
K187A	6.38 ±0.17	63.67 ±7.13
F257A	4.759 ±0.14	38.33 ±13.70 ***
F292A	4.44 ±0.37 **	89.50 ±4.50
K92A	5.01 ±0.16	52.33 ±6.98 **
E185A	5.69 ±0.12	62 ±5.86
L193A	5.642 ±0.05	70.67 ±10.71
S196A	6.10 ±0.05	56.33 ±5.24 **
A291F	5.37 ±0.08	54 ±9.29 **

(Data are shown as mean ± SEM of at least three independent experiments performed in technical replicates. All data were analyzed by two-sided, one-way ANOVA by Dunnett's multiple test compared with WT. *$P < 0.05$, **$P < 0.01$ and ***$P < 0.001$ were considered statistically significant.)
